# The Spread of Lung-disease in the German Empire during the Year 1896

**Published:** 1899-06

**Authors:** 


The Spread of Lung-disease in the German Empire During the Year 1896.—In the statistical year 1896 the lung-disease was considerably more destructive than in the preceding. The total number of cattle affected was 1608. The cases are distributed over seventy communities, including 185 farms in seven different States, namely, Prussia, Bavaria, Saxe-Weimar, Brunswick, Anhalt, and Reuss. The contagion was spread widest in the governmental departments—Magdeburg (113 farms affected); Cologne (49 farms); Potsdam (26 farms); Düsseldorf (15 farms). To the districts strongly affected must be added Wolmirstadt, Wauzleben, Neuhaldensleben, Osthavelland, and Euskirchen. Of every 10,000 cattle in the empire 0.92 were afflicted; for the different States this number varies from 0.15 (in Reuss) to 0.01 (in Bavaria). Eighteen cattle died of the disease; 1754 were killed by order of the sanitary police, and 946 at the request of the owners; 23.1 per cent. of those killed by police orders and 75.4 per cent. of those killed by request of the owners were found free from lung-disease at the post-mortem. The total loss of cattle in thus fighting the contagion amounted to 2737 animals. The greatest spread geographically took place during the first three months of the year. The second three months show the greatest loss.

From abroad the following statistics have been received: Belgium, 2 cases; France, 380; Great Britain, 9; Italy, 9. In Austria only one place was affected at the close of the year. Causes of outbreaks: In one case the disease was brought in from Bohemia to Bavaria on an animal imported for breeding purposes. In Prussia numerous infections took place through the cattle trade.

The cases were discovered mostly in consequence of notice from the owners, in some cases on inspection of the meat. The duration of incubation varied from forty-one to sixty days.

Tuberculin injections were made in twenty-four herds; of these twelve were affected and twelve did not react. Of the herds affected six had already in part or completely been inoculated when the contagion appeared. Of the six inoculated herds, embracing 224 inoculated cattle, eight animals fell sick; of the seventy-five which had not been inoculated seven fell sick. In the six herds which were not inoculated until after the outbreak of the contagion twenty-four out of 544 were taken sick; after the inoculation of 519 eighteen of them were affected.

As indemnity for the 2219 cattle the State paid out 401,939 marks and 91 pfennigs.— *Idem.*

